# Hybrid Triboelectric Nanogenerators: From Energy Complementation to Integration

**DOI:** 10.34133/2021/9143762

**Published:** 2021-02-24

**Authors:** Lingjie Xie, Ningning Zhai, Yina Liu, Zhen Wen, Xuhui Sun

**Affiliations:** ^1^Institute of Functional Nano and Soft Materials (FUNSOM), Jiangsu Key Laboratory for Carbon-Based Functional Materials and Devices, Soochow University, Suzhou 215123, China; ^2^Department of Applied Mathematics, Xi'an Jiaotong-Liverpool University, Suzhou 215123, China

## Abstract

Energy collection ways using solar energy, wave, wind, or mechanical energy have attracted widespread attention for small self-powered electronic devices with low power consumption, such as sensors, wearable devices, electronic skin, and implantable devices. Among them, triboelectric nanogenerator (TENG) operated by coupling effect of triboelectrification and electrostatic induction has gradually gained prominence due to its advantages such as low cost, lightweight, high degree of freedom in material selection, large power, and high applicability. The device with a single energy exchange mechanism is limited by its conversion efficiency and work environment and cannot achieve the maximum conversion of energy. Thus, this article reviews the research status of different types of hybrid generators based on TENG in recent years. Hybrid energy generators will improve the output performance though the integration of different energy exchange methods, which have an excellent application prospect. From the perspective of energy complementation, it can be divided into harvesting mechanical energy by various principles, combining with harvesters of other clean energy, and converting mechanical energy or various energy sources into hydrogen energy. For integrating multitype energy harvesters, mechanism of single device and structural design of integrated units for different application scenarios are summarized. The expanding energy harvesting efficiency of the hybrid TENG makes the scheme of self-charging unit to power intelligent mobile electronic feasible and has practical significance for the development of self-powered sensor network.

## 1. Introduction

As a sign of the new era, multifunctional electronic devices are changing the way people perceive the world and improving the efficiency of people's life [1–3]. Because electronic equipment cannot live without electricity, the process of human informationization and intelligentization depends on extensive energy supply and powerful electric power support [4, 5]. However, with the widening consumption of traditional fossil fuel and the improvement of awareness of environmental protection on which we live, higher requirements are put forward for global energy resources. On the one hand, there is an urgent need to change the energy structure from scarce, polluted, and nonrenewable mineral resources into abundant, environmentally friendly, and renewable green energy [6, 7]. On the other hand, the waste of environmental energy and disordering energy transportation induce low energy efficiency, which exist in both traditional energy and renewable energy [8]. Therefore, the unreasonable energy structure and the low energy efficiency lead to the current development dilemma.

From the perspective of the long-term economy and environment, green energy from our living environment is an ideal choice for future electronic devices [9, 10]. Therefore, green energy, including hydropower, wind power, thermal energy (including land source and water source), bioenergy, and tidal energy, has gradually attracted people's attention [11]. Among them, the electromagnetic generator provided the basis for large-scale use of electricity, which has brought people from the age of steam engine into the age of electrification and great development for human [12, 13]. Solar cells [14, 15], thermoelectric generators [16], and biofuel cells [17] give people a glimpse of the future of clean energy. Although they are not affected by energy shortages, they are too dependent on natural conditions, such as solar, wind, and heat resource, making it difficult for them to be utilized in a variety of environments and high cost of investment and maintenance. And mechanical energy, as one of the most readily available energies in daily life, such as human movement, has many advantages, such as availability, continuity, and independence [18, 19]. But because of its disperse form, small energy density, and low frequency, it is often neglected and wasted. Due to the progress of nanotechnology, triboelectric nanogenerators (TENG) make it possible to harvest mechanical energy conveniently and efficiently [20–24]. TENG can realize energy harvesting triggered by low-frequency mechanism and can make use of various types of mechanical energy effectively. TENG has a wide range of applications, such as wearable power supply [25–27], motion tracking [28], health monitoring [29, 30], and Human Machine Interface (HMI) [31–33].

These energy devices are often not appropriate together attributed to weather conditions, equipment environment, and other factors. Researchers have developed a variety of integrated energy devices to acquire from diversity of power sources, especially based on TENG [34, 35], which has the advantages of lightweight, eco-friendly, and widespread availability. The blended power system could coincidentally or separately obtain various energies to ensure the continuous operation of devices or sensors under any conditions. Compared with a single type of generator, the hybridized generator has higher output power, higher machine-electric conversion efficiency, and better environmental adaptability [36, 37]. It can solve the power supply problem of some electronic devices that required higher power.

**Figure 1:**
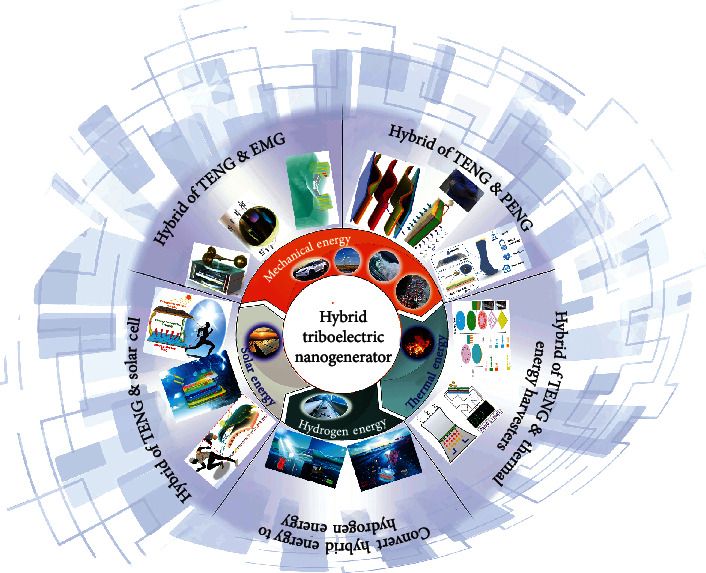
Schematic illustrations of the hybrid triboelectric nanogenerator (TENG) with different energy harvesters.

In this review, hybrid triboelectric nanogenerator (TENG) with different energy harvesters is introduced from energy complementation to integration. As introduced in Figure 1, the main latest energy harvested by hybrid energy cells includes mechanical energy, solar energy, thermal energy, and hydrogen energy. As for mechanical energy, the common integrated structures are TENG with the electromagnetic generator (EMG) and TENG with piezoelectric nanogenerator (PENG) to expand the efficiency of mechanical energy. In the energy complementation from thermal energy, TENG can be combined with the pyroelectric nanogenerator and thermoelectric generator for varying temperature. Insolation energy can be transformed to electrical energy by photovoltaic cells or to hydrogen energy by photoelectrochemical water splitting. Both methods can be combined with TENG to extend working hours. TENG can store mechanical energy as hydrogen energy by driving electrochemical water splitting. The hybrid TENG will provide a stable development foundation for self-charging energy packets and self-powered sensors.

## 2. Integration of Multifarious Mechanical Energy Harvesters

### 2.1. Hybrid of TENG and EMG

#### 2.1.1. Theoretical Distinction of TENG and EMG

**Figure 2:**
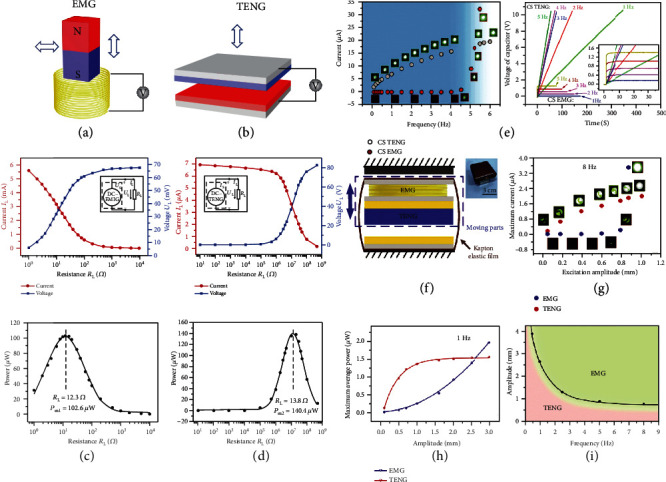
Theoretical comparison of EMG and TENG: (a) the electromagnetic induction; (b) output characteristics with the different external resistive loads of the rotating DC-EMG; (c) the triboelectrification; (d) output characteristics with the different external resistive loads of the rotating DC-TENG [39] (reproduced with permission. Copyright John Wiley and Sons, 2014); (e) output performance comparison between CS-mode EMG and TENG at low frequency [50] (reproduced with permission. Copyright American Chemical Society, 2016); (f) schematic and photograph of the integration of EMG and TENG [51] (reproduced with permission. Copyright Elsevier, 2019); (g) maximum current with different excitation amplitudes at 8 Hz which is driven by the EMG and TENG; (h) maximum average power comparison of EMG and TENG with different amplitudes at 1 Hz; (i) applicable frequency and amplitude of TENG and EMG.

The electromagnetic generator (EMG) is developed by using the Faraday law of electromagnetic induction, which was proposed in 1831. After more than one century of development, EMG provides the basis for large-scale use of electricity, which has brought people from the age of steam engine into the age of electrification and great development for human. Mechanical energy, as one of the most common energies in the human living environment, has the characteristics of diversity, independence, accessibility, and universality [38]. The traditional EMG is a major mechanical energy supply equipment, and it mainly makes use of the relative motion of the magnet and coil to change the magnetic flux through the coil and generate the induced electromotive force. As a result, in a closed coil, an induced current is generated, as shown in Figure 2(a).

The magnitude of induced electromotive force is ascertained by Faraday's law of electromagnetic induction, as follows [39]:

(1) $$\begin{array}{c} E=-N\frac{d\Phi }{dt}=-N\frac{B\cos\theta dS}{dt}=-N\frac{B∙dS}{dt}, \end{array}$$

where *E* is the induced electromotive force, *B* is the magnetic induction intensity, *Φ* is the total magnetic flux through the coil, *θ* is the angle between the normal of the loop element and the magnetic induction intensity *B*, *S* is the coil area, and *N* is the number of turns in a coil. Therefore, in order to design high-performance EMG, it is necessary to optimize these parameters to obtain a higher induced electromotive force.

Unlike EMG, TENG is another mechanical energy harvester, which is relatively simple in design, and does not require high-frequency mechanical input. The mechanism of TENG is through the coupling effect of the contact electrification and the electrostatic induction. The contact electrification is ascribed to the gain and loss of electrons between two different materials which have relative polarity, inducing the triboelectric charge on the surface by physical contacting. Recently, Xu et al. [40–42] and Lin et al. [43] proceeded with many theoretical studies to further explore triboelectric electrification and found that electron transfer is the main process of contact electrification rather than ion transfer. The electrostatic induction is the rearrangement of electronics in the electrode attributable to the domination of close electrons and totally equilibrium of the electric field. The electrostatic field leads electrons to move between two objects by an outer loop, thereby transforming mechanical energy into electrical energy, as presented in Figure 2(b). There are four main fundamental modes [44–49], which have the electric current caused by the polarization field [39]:

(2) $$\begin{array}{c} I=N\frac{∆Q}{∆T}, \end{array}$$

where *N* is the segment number which is on behalf of the number of charge transfers per unit time between two objects and ∆*Q*/∆*T* is the charge transfer rate in each segment. From these two formulas, TENG can be seen as a current source and EMG can be seen as a voltage initiator. Furthermore, these parameters are related to mechanical movement, and these two harvesters are all proved to be the main devices for collecting mechanical energy.

For theoretical distinction and the performances of EMG and TENG, Zhang et al. chose the rotational mode EMG and rotation-sliding mode TENG to analyze [39]. The rotation-sliding mode TENG was intended of four sections and 12.4 cm^2^ of friction area. The rotational mode EMG was designed with 12 turnings in a coil, 0.05 T of the magnetic flux density, and 25.3 cm^2^ of the coil area. At the same time, they have the identical angular velocity which is 20*π* rad/s. Since the output of both TENG and EMG is alternating current (AC) which is not easy to directly supply power to electronic equipment, a brush was used in this work to switch AC into direct current (DC) output. Figure 2(c) shows output performances (DC) with a distinctive outer resistance of the EMG. The voltage increases with the decreasing resistance, at the same time as the current shows an opposite trend, but when the resistance is substantially large, the voltage tends to be saturated which is 67.6 mV. The output power achieves a maximum of 102.6 *μ*W. The small matching impedance (12.3 *Ω*) of EMG indicates that the EMG has a small internal resistance. Figure 2(d) shows the resistance dependence of output performances of the DC-TENG, from 10 *Ω* to 500 M*Ω*. The same as the EMG, the output voltage increases with the increase of the resistance and reaches the maximum value of 140.4 *μ*W. Hence, the TENG with high matching impedance (13.8 M*Ω*) could be informed as a current source attributed to its high internal resistance.

In addition to the differences in the intrinsic utility of TENG and EMG, they also have a specific advantage range in different working frequencies and amplitudes. According to the figures of merit (FOMs) of contact-separation mode TENG (CS-TENG), which is typical within four modes, the basic systematic comparisons based on the CS mode are analyzed. Most of the mechanical energy in the environment is mainly below 10 Hz, such as human motion, water wave, tide, and wind. Because the energy acquisition frequency band of the energy harvester composed of a single energy exchange mechanism is narrow, which is not conducive to collect the wide-frequency mechanical energy, it is important to analyze the collection frequency of TENG and EMG. Zi et al. described a relative work of low-frequency mechanical energy garnering based on contact-separation mode TENG and EMG [50]. In Figure 2(e), at the threshold frequency (*f*_th_) of ~5 Hz for CS-TENG and EMG, it contributes to equal average power densities. When the frequency range is below *f*_th_, TENG can easily light up the LED with high voltage and low current, while the EMG needs to get the required voltage and current by achieving a minimum frequency (~4.5 Hz for the EMG). Meanwhile, due to the limit of low voltage of EMG, the saturation voltage of charging capacitors is very low comparing with TENG. This means that most of the low-frequency mechanical energy cannot be stored and wasted by EMG. In summary, TENG with high open-circuit voltage (*V*_OC_) and low short-circuit current (*I*_SC_) is dominant to harvest <5 Hz frequency (CS-mode) mechanical energy.

Environmental vibration energy is a kind of renewable and clean energy with abundant reserves and wide distribution. Through the TENG and EMG, the mechanical vibration energy in the environment can be converted into electric energy to supply power for wireless sensor network nodes, which is an effective solution to break the limitation of traditional power supply mode. For the complex and changeable environment vibration energy, Zhao et al. studied the influence of motion amplitude on two kinds of generators and revealed the dominant amplitude range [51]. Figure 2(f) shows a hybrid structure of EMG and TENG to work at the same amplitude and frequency. At 8 Hz, there is a threshold amplitude of 0.87 mm for the same maximum current of EMG and TENG in Figure 2(g). Below 0.87 mm, the slow growth of the maximum current in EMG results in only weak LED lighting, while TENG is able to light a LED at brighter spots at 0.1 mm amplitudes. So the amplitude below 0.87 mm is the dominant amplitude region of TENG compared to EMG at 8 Hz. TENG has certain advantages at small frequencies; then, the threshold amplitude is a little higher than that at large frequencies. As shown in Figure 2(h), the experimental results demonstrate that the TENG has a higher maximum average power than that of the EMG within 2.6 mm at 1 Hz. What is more, Figure 2(i) summarizes the intersection of these two maximum average output characteristics at different frequencies. The amplitude of the edge declines with the growing frequency, which means the advantage scope of TENG is narrowing. The results indicate that TENG has a predominant extent at small amplitude even at high frequencies, and the advantage scope is especially obvious in low frequencies.

#### 2.1.2. Hybrid Operation Driven by Wind Energy

Wind power is considered one of the bright renewable sources of energy, because it is a free natural resource and is conceptually relatively easy to control and use [52, 53]. Reducing the pollution of the ecological environment in the production and consumption process as much as possible, wind energy, as an efficient and clean new energy, has great potential for development. Last year, the world's wind power capacity exceeded 651 GW, and installed capacity is steadily rising [54, 55]. In the traditional EMG-based wind turbine with large current and strong stability, adding the part based on TENG can effectively utilize the energy of low wind speed and generate high voltage.

**Figure 3:**
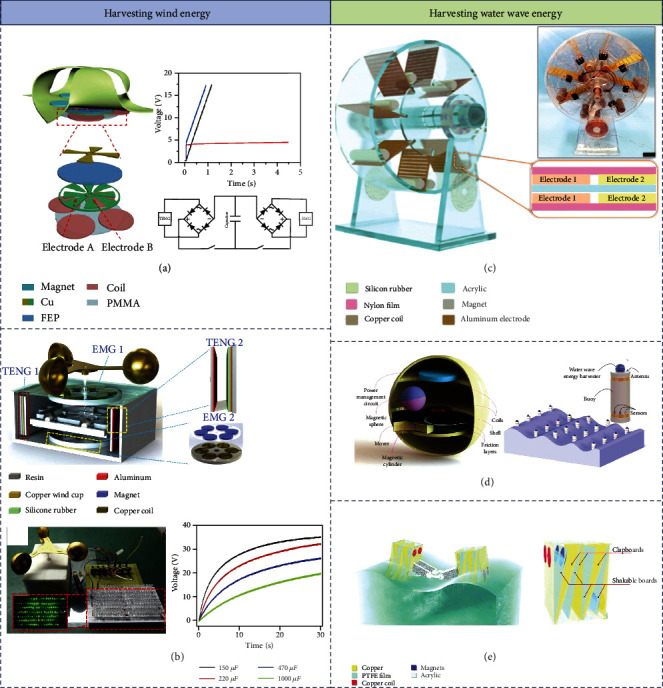
Hybrid operation driven by EMG and TENG for harvesting wind energy and water wave energy: (a) a hybridized design of pinwheel driven by wind that based on sliding-mode TENG [56] (reproduced with permission. Copyright Elsevier, 2019); (b) a hybrid nanogenerator driven by wind that based on contact-separate mode TENG [57] (reproduced with permission. Copyright Elsevier, 2020); (c) a full-packaged waterwheel-like rolling triboelectric–electromagnetic hybrid nanogenerator (TEHNG) [58] (reproduced with permission. Copyright Elsevier, 2019); (d) a hybridized water wave energy harvester (WWEH) that based on a magnetic sphere [59] (reproduced with permission. Copyright American Chemical Society, 2019); (e) an optimized inner topological structure of a hybrid nanogenerator [60] (reproduced with permission. Copyright John Wiley and Sons, 2019).

Guo et al. designed a novel pinwheel that consisted of the TENG part and EMG part for harvesting air-current power, as displayed in Figure 3(a) [56]. In the TENG part, there is a rotator with radial-arrayed copper sections as one friction layer and a stator with fluorinated ethylene propylene (FEP) cover in the role of another friction layer and copper electrode layers. For the EMG part, four groups of small magnets were on the vane of the pinwheel, and relatively four copper coils were attached to the back of the stator. When the rotator is driven by wind, alternating current (AC) is generated by these two individual parts. The AC signals are converted into DC output by two rectifier bridge circuits and then charge the capacitor. The capacitor (1 *μ*F) can be charged to 17.3 V by TENG and composite output at the speed of 6 m/s but only get to 3.8 V by EMG part with high beginning charging speed, since the charging rate of the capacitor is determined by the output current, which is attributed to the high current of EMG. Because the final voltage is subject to the output voltage, the TENG conduces to the final charging voltage.

Furthermore, to solve the problem that the output power of energy devices is too small to meet the power demand of wireless monitoring systems, this chapter proposes a hybrid wind energy reaper reached from triboelectric-electromagnetic power generation technology coupling. Fan et al. expressed a hybrid nanogenerator (TEHG) to achieve high output, high conversion efficiency, and wide frequency response [57]. In Figure 3(b), the Cu cup on the top of the skeleton could be utilized to harvest wind energy and offers rotational motion for the power generator set. The TENG unit utilizes silicone rubber as the triboelectric negative material and the aluminum layer as the positive material. The straight-up contact-separation structure has been designed to avoid the loss of triboelectric material covering and to realize the stability of the mechanical property and electrical output property of the device. The EMG unit is designed with a rotating structure between the coil and the magnet, and the collected wind energy is converted into rotating kinetic energy through the structure to generate induced current. The TEHG could brighten 165 green LEDs and charge a capacitor of 1000 *μ*F to 19.8 V in 30 s.

#### 2.1.3. Hybrid Operation Driven by Water Wave Energy

The total area of the earth's oceans is about 360 million square kilometers, accounting for about 71 percent of the earth's surface area, so the extremely rich water resources of the ocean are offering supports to the growth rate of ocean emerging industries [61, 62]. Water wave energy refers to the kinetic and potential energy of waves at the ocean surface, which could carry on any period, twenty-four hours, different climate, and cold or heat. It is estimated that the power generated by waves hitting the coastline around the world is about 2 TW. If the wave energy is collected in the open sea, the world energy future will be quite broad and bright [63, 64]. According to the utilization mode of wave energy, wave energy generation can be roughly divided into vertical movement using wave energy, horizontal movement using wave energy, water pressure using wave, water point using wave motion, and so on [65]. However, EMGconsisted of magnets, metal, coil and turbine, and can't easily float on the surface unless supported by a floating platform, which is expensive and technically difficult. Coil and magnet are vulnerable to water corrosion without a complete package. And the turbines are inefficient at tiny frequencies and the moving manners of irregular water waves. These challenges make the tiny energy collection efficiency and high cost. However, TENG is more adaptable to irregular and random mechanical movements than EMG. At present, it is a reliable method to harvest wave energy, which is irregular and has low frequency, by combining TENG and EMG. This chapter mainly analyzes the efficient and durable composite structure of the current design and provides ideas for the design and production of some devices that can realize large-scale commercial exploitation and development of marine energy.

Firstly, environmental conditions such as humidity could affect the performance of TENG and EMG. So, Yang et al. invented a fully packaged water-wheel rolling triboelectric–electromagnetic hybrid nanogenerator (TEHNG), which has harvested water wave energy in the wicked conditions [58]. As shown in Figure 3(c), a rolling free-standing mode triboelectric nanogenerator (RFS-TENG) was fabricated by using silicone rubber-magnet rods and nylon thin-film laminated electrodes, which decreases damage and increases durability. An EMG is designed in the system by combining with copper coils. The cylindric structure is more easily driven by water flow than other angular structures.

Secondly, a spherical independent layer mode TENG is a precious approach to enhance the water wave energy harvesting from low-frequency water wave motion. A hybridized water wave energy harvester (WWEH) developed on a magnetic sphere was exhibited by Wu et al. [59]. In Figure 3(d), for the TENG part, a friction unit is sliding on a solid layer, which is driven by the rolling magnetic sphere. Meanwhile, the rolling magnetic sphere and two coils convert the moving mechanical energy to electricity. The WWEH is successfully driven scattered, self-driven sensors for environmental observation, because of its high output.

However, the ball-shell structure still cannot achieve high output power, because of the electrostatic shield effect and inadequate contact.. Wang et al. proposed a hybrid system based on an optimized cubic structured unit for water wave energy harvesting [60]. As shown in Figure 3(e), the TENG part consists of two parts, four separate acrylic chambers and four shakable boards. The polytetrafluoroethylene (PTFE) film as triboelectric material attached to the board can contact fully with Cu electrodes secured on the inner walls of each chamber. The EMG part consisted of two pairs of magnets embedded in the shakable block and two pairs of copper coils installed on the outer walls of the chambers. Besides, this article concluded the optimal operating frequency ranges for TENG and EMG.

### 2.2. Hybrid of TENG and PENG

#### 2.2.1. Theoretical Distinction and Hybridized of TENG and PENG

Maxwell introduced the displacement current, *∂ ***D**/*∂t*, in Ampere's law, to satiate the command of conservation of charge, so that the electricity and magnetism can be unified, in which D is the electric displacement vector. He proved the correspondence of electricity and magnetism on this basis [66]. In 2017, for the first time, Wang extended the statement of displacement and import *P*_*S*_ in D to drive the nanogenerator (NG) output; *P*_*S*_ is the polarization created by the electrostatic charge, which is derived mechanical trigger and different from the electric field of dielectric polarization *P* [67, 68]. These charges from piezoelectric polarization and triboelectrification whether or not have an outer applied electric field. Maxwell's total displacement current density is regarded as

(3) $$\begin{array}{c} J_{D}=\frac{\partial D}{\partial t}=\varepsilon \frac{\partial E}{\partial t}+\frac{\partial P_{\text{S}}}{\partial t}, \end{array}$$

where D is the displacement field, *ε* is the dielectric constant of the medium, and *E* is the electric field. In this formula, the first term is the electric field that changes over time, whilst the second phrase is the support of surface polarization and the start of nanogenerators. In piezoelectric nanogenerators (PENG), the surface polarization derives from the piezoelectric polarization charge produced by the applied strain [69], while in TENG, the triboelectric charge generated only by the touch between two distinctive objects will form the surface polarization over time [20]. The displacement current is a surface integral of *J*_*D*_:

(4) $$\begin{array}{c} I_{D}=\int J_{D}∙ds=\frac{\partial Q}{\partial t}, \end{array}$$

where *Q* is the full free charge on the electrode. It can be seen from this equation that the internal circuit of the nanogenerator is dominated by displacement current, while the current observed by the external circuit is the capacitive conducting current. Displacement current is the internal physical core of current generation and the internal driving force of the current generation, while the capacitive conducting current in the external circuit is the external manifestation of displacement current [70].

**Figure 4:**
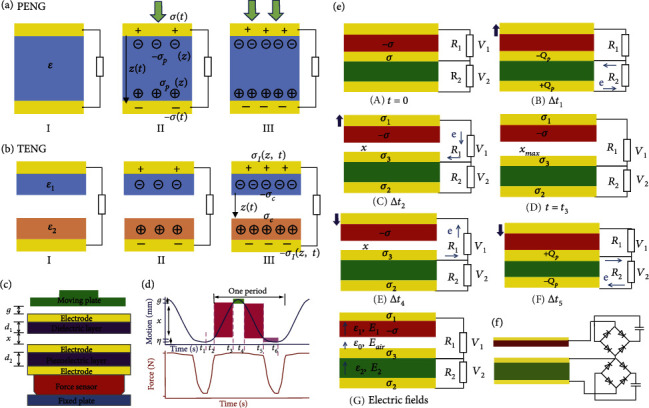
Theoretical comparison and of EMG and TENG and theoretical analysis of the contact-mode hybrid nanogenerator (HG): (a) the working principle of piezoelectric nanogenerator (PENG); (b) the working principle of TENG [71]; (c) schematic diagram of the device of HG; (d) the curves of motion-time and force-time; (e) the working mechanism of the HG; (f) equivalent circuit of the HG to charge two capacitors [72] (reproduced with permission. Copyright John Wiley and Sons, 2016).

Figure 4(a) establishes the working mechanism of PENG [67]. An insulated piezoelectric material covers the upper and underside with two electrodes (I). Vertical mechanical deformation causes a piezoelectric polarized charge to be generated (II), and a growth in external pressure causes a larger polarization charge density (III). The electrostatic potential generated by a polarized charge is equitable due to the move of electrons from an external load from one electrode to another. The density of the piezoelectric polarized charge on the surface is **σ**_*p*_(**z**), which can be raised by rising the applied pressure and the corresponding charge density of the free electron in the electrode is **σ**(**t**). Because of the existence of external resistance *R*, the output association of the PENG is [71]

(5) $$\begin{array}{c} RA\frac{d\sigma }{dt}=\frac{z\left[\sigma_{p}\left(z\right)-\sigma \left(t\right)\right]}{\varepsilon }, \end{array}$$

where *A* is the electrode area. *z* is a function of time *t* in the event of the strain applying relatively slowly.

For the TENG, the electrostatic charges are generated by the contact of two different materials, and the time-varying electric field drives the flow of electrons in the external circuit. For example, the contact-separation mode of TENG consists of two interstice-separated dielectric layers with electrodes located on the upper and underside of each layer. If the dielectric constant of two dielectric layers is *ε*_1_ and *ε*_2_ and the thickness is *d*_1_ and *d*_2_ and the surface charge density from the friction is *σ*_*c*_(*t*) and the density of free electrons is *σ*_*I*_(*z*, *t*) on the surface of the electrode, then the electric field in the two layers and the gap is *E*_*z*_ = *σ*_*I*_(*z*, *t*)/*ε*_1_ and *E*_*z*_ = *σ*_*I*_(*z*, *t*)/*ε*_2_, respectively. The potential decrease between the two electrodes is as follows [67]:

(6) $$\begin{array}{c} V=\sigma_{I}\left(z,t\right)\left[\frac{d_{1}}{\varepsilon_{1}}+\frac{d_{2}}{\varepsilon_{2}}\right]+z\frac{\left[\sigma_{I}\left(z,t\right)-\sigma_{c}\right]}{\varepsilon_{0}}. \end{array}$$

Considering the current transport process in external circuits caused by triboelectric fields, due to the presence of external loads, the current output equation of TENG, according to Ohm's Law, is

(7) $$\begin{array}{c} RA\frac{d\sigma_{I}\left(z,t\right)}{dt}=\frac{z\sigma_{c}}{\varepsilon_{0}}-\sigma_{I}\left(z,t\right)\left[\frac{d_{1}}{\varepsilon_{1}}+\frac{d_{2}}{\varepsilon_{2}}+\frac{z}{\varepsilon_{0}}\right] \end{array}$$

where *z* is a function of the time *t* that depends on the dynamic procedure of operating force.

Since the PENG and TENG have similar structure characters and matching impedance and the couple two could transform mechanical energy into electricity, they have the capability to be combined into one structure to increase the electrical output and energy efficiency. Most importantly, hybrid generators (HG) that consisted of TENG and PENG are suitable for working in contact mode. Understanding the fundamental mechanisms and then designing a theoretical model are beneficial to optimize the HGs, which are studied by Chen et al. [72]. Firstly, *g* presents the sundering length among the driving plate of tester and electrode 1, and *x* presents another sundering length among the triboelectric surface and electrode 2, as displayed in Figure 4(c). *η* displays the movement of the triboelectric surface and the piezoelectric surface. In Figure 4(d), one period can be divided into five stages from Δt_1_ to Δ*t*_5_. Figure 4(e) shows the specific charge changes at different stages. Firstly, the dielectric layer and electrode 2 contact with each other, and because of the triboelectric effect, each layer has opposite triboelectric charges, which reach a saturation state (surface charge density, *σ*). During the time of Δ*t*_1_, when the pressure starts to release, the moving plate starts from *η*_max_ to *η*_0_. The squeezing stress of PENG part is reducing until 0 (at *t*_2_), and piezoelectric potential is generated which drives electrons to move at the external connection reversely. During the time of Δ*t*_2_, when the moving plate goes up, these two contact layers begin to separate and the gap distance *x* is increasing to *x*_max_. As a result, the electric field is redirected ascribed to the electrostatic induction. At *t*_3_, when *x* reaches *x*_max_, the transferred charges will reach their maximum values without generating electrical output. In the period of Δ*t*_4_, *x* decreases from the uttermost to zero and drives the electrons to recede. After that, when the charged surface has full access to electrode again, charges of the triboelectric layer and electrode return to its original state (*t*_5_), and the output current decreases to zero. In the period of Δ*t*_5_, the piezoelectric potential is produced by compressing and results in electrons flowing from electrode 3 to electrode 2. On account of the AC signal of TENG and PENG which is difficult to charge electric devices directly, capacitors are associated by a double-bridge rectifier loop.

#### 2.2.2. Hybrid Operation of TENG and PENG

**Figure 5:**
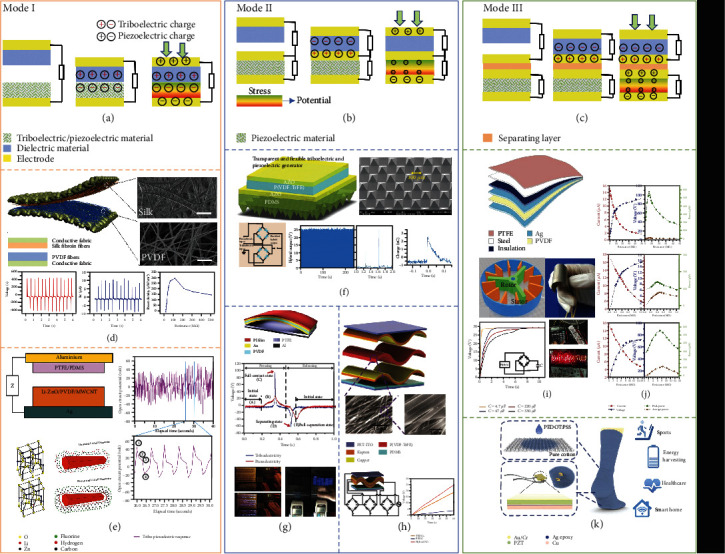
Hybrid operation of TENG and PENG in three modes: (a) mode I which shares a pair of electrodes; (b) mode II which shares one electrode; (c) mode III which works independently; (d) a textile-based wearable hybrid triboelectric-piezoelectric nanogenerator (TPNG) [77] (reproduced with permission. Copyright Elsevier, 2018); (e) a tribo-piezo hybrid nanogenerator (PTENG) with PVDF/MWCNT/LieZnO as the piezoelectric constituent and PTFE/PDMS as the triboelectric constituent [78] (reproduced with permission. Copyright Elsevier, 2019); (f) a transparent, flexible, and stretchable triboelectric and piezoelectric generator (TPG) [79] (reproduced with permission. Copyright Royal Society of Chemistry, 2016); (g) an arched-shaped cooperative operation of PENG and TENG [80] (reproduced with permission. Copyright Springer Nature, 2013); (h) a wave-shaped hybrid nanogenerator based on P(VDF-TrFE) nanofibers [81] (reproduced with permission. Copyright Royal Society of Chemistry, 2016); (i) a hybrid device based on an induced compound method [82]; (j) impedance characteristics for TENG, PENG, and hybrid generator (reproduced with permission. Copyright Elsevier, 2020) (k) a self-powered and self-functional sock by hybrid integrating piezoelectric and triboelectric hybrid mechanisms [83] (reproduced with permission. Copyright American Chemical Society, 2019).

From the characteristic of TENG and PENG above, the coherent orientation of piezoelectric polarized charges and triboelectric charges could build up energy efficiency from mechanical energy to electricity. Attributed to wearable electronic devices playing an important role in the development of the Internet of Things (IoT), flexible power supply devices with high flexibility came into being [73–75]. Normally, the energy harvester applied to the wearable device converts the mechanical energy in the environment with other energy sources into electrical energy that can continuously power the wearable device and the sensor. Therefore, more and more different TENGs and PENGs have been studied, which can be divided into two types: wearable and implantable, due to their advantages of flexibility, portability, and low cost. With the development of microelectronic technology and material science, a variety of piezoelectric materials, triboelectric materials, and their processes have been realized. The power density and charging effect of the composite mechanism can be generally higher than those of one single mechanism. Meanwhile, structural design plays a crucial role in the composition of output. According to the structural design with different shared parts, there are three modes of hybrid coupling [76]: TENG and PENG share duad electrodes (mode I), TENG and PENG utilize one shared electrode (mode II), and TENG and PENG work independently (mode III), as exhibited in Figures 5(a)–5(c).

In mode I, it is the simplest structure based on the materials with both the piezoelectric and triboelectric effects. Combining the advanced energy harvesting device with the traditional textile technology, the hybrid nanogenerator based on fiber has emerged, which provides the mechanical energy harvesting and multifunctional self-power supply for the intelligent textile, while maintaining the flexible and universal wearable power platform. As shown in Figure 5(d), Guo et al. present a textile-based hybrid triboelectric-piezoelectric nanogenerator (TPNG) [77]. Silk fibroin nanofibers and poly(vinylidene fluoride) (PVDF) nanofibers are electrospun on the conductive fabrics, respectively. To optimize the reaction between two effects and enhance the output performance, the current directions of triboelectricity and piezoelectricity are accordant by finding the reasonable polarization direction. The output performance (*V*_OC_, *I*_SC_, and power density) of fabricated TPNGs is 500 V, 12 *μ*A, and 0.31 mW/cm^2^, which shows high power levels, respectively. PVDF is used as the major part of piezoelectric panel and also as triboelectric layer. Furthermore, in order to improve the overall output, it is a way to improve the polarization performance by surface modification. Surface-modified Li-ZnO nanowires are induced into PVDF to increase its piezoelectric response, and MWCNT was induced into the composites of Li-ZnO/PVDF by Chowdhury et al. [78]. The *V*_OC_ response of finger press is shown in Figure 5(e). MWCNT creates a 3D network in the polymer matrix and increases the conductivity of composites, which ease off electron flow during triboelectric/piezoelectric actions. In the triboelectric component, PTFE/PDMS introduces PTFE particles into PDMS to improve the triboelectric effect, and PVDF/MWCNT/Li-ZnO are the corresponding layer of friction material.

In mode II, the hybrid nanogenerator is not using piezoelectric material as a triboelectric layer, which has two separate parts. The upper part of the triboelectricity can adopt the single-electrode mode, adding more working modes, suitable for harvesting human body mechanical movement. Wang et al. designed a pellucid, flexible, and wearable hybrid energy harvester, as shown in Figure 5(f) [79]. The device from bottom to top is as follows: the triboelectric layer PDMS, the electrode layer Al:ZnO(AZO) below, the piezoelectric layer P(VDF-TRFE), and the upper electrode layer AZO are closely bound together due to process reasons. Since all the materials are biocompatible and nontoxic, it is suitable for wearing devices in direct contact with the skin. Because the piezoelectric and triboelectric mechanisms work together in a single pressing and releasing cycle and high output power can be generated, Jung et al. demonstrated a prototypical PENG/TENG hybrid generator based on an arch shape [80]. This generator is composed of two layers: an Au/PVDF/Au layer on the top as PENG unit and a PTFE/Al layer on the bottom as TENG unit which contact and separate with the Au electrode to generate electrons. The graph of the piezoelectric and triboelectric output voltages explains the mechanism of the hybrid generator. The *V*_OC_ of TENG is higher than that of PENG unit, and a delay in the peaks occurs in the second part of the output curve, which means the piezoelectric charges are returning slower than the triboelectric charges during releasing due to the residual strain in the PVDF. The output power successfully lights up 600 LEDs by giving a 0.2 N mechanical force. As the polarization of the piezoelectric material occurs, the output of TENG and PENG part can be enhanced if electric potential difference at both sides' electrodes of PENG is larger. It can come true through rational structure design and material selection. Chen et al. presented a wave-shaped hybrid nanogenerator based on P(VDF-TrFE) nanofibers [81]. In Figure 5(h), the hybrid nanogenerator is designed as three parts, including the upper TENG (PET-ITO/Kapton-Cu), the PENG (Cu/P(VDF-TrFE)/Cu), and the lower TENG (PET-ITO/PDMS/Kapton-Cu). After multiple contacts, press, release, and separations, the top electrode has a positive potential as the bottom electrode has a negative potential. At the same time, because of the electrostatic induction, the potential distribution is inverted on these two electrodes. In conclusion, more electrons are flowing in the external circuit which is inferred from the larger potential difference on each part of the hybrid nanogenerator.

In mode III, PENG and TENG parts are made separated by the addition of an insulating layer, compared with mode II. It is more convenient to design for boosting the average output power of every part of hybrid systems and making use of piezoelectric units to compensate for the attenuation of triboelectric output. Bu et al. demonstrate a force-lured composite way to obtain a hybrid nanogenerator. Firstly, force lures frictions to produce triboelectric power and then arouse girder deflections to make the self-oscillation immediately and produce piezoelectric power. The structure and output characteristics of the hybrid instrument have been displayed in Figure 5(i). In Figure 5(j), the hybrid nanogenerator achieves Watt-level average power in low frequency, compared with TENG and PENG, respectively. As shown in Figure 5(k), through the integration of lead zirconate titanate (PZT) piezoelectric sensor and poly(3,4-ethylene dioxythiophene) polystyrene sulfonate- (PEDOT: PSS-) coated TENG textile, a self-driven and multifunctional sock is invented.

## 3. Integration of Mechanical and Thermal Energy Harvesters

### 3.1. Theoretical Comparison of TENG, Pyroelectric Nanogenerator, and Thermoelectric Generator

From a long-term economic and environmental point of view, green energy from the environment we live in is the ideal choice for future wearable electronic devices. There is a large amount of wasted heat energy in production and life. How to harvest this energy and transform it into electricity we need has attracted more and more attention. Meanwhile, TENGs also unavoidably produce heat deriving from the friction that develops from their sliding movement.

**Figure 6:**
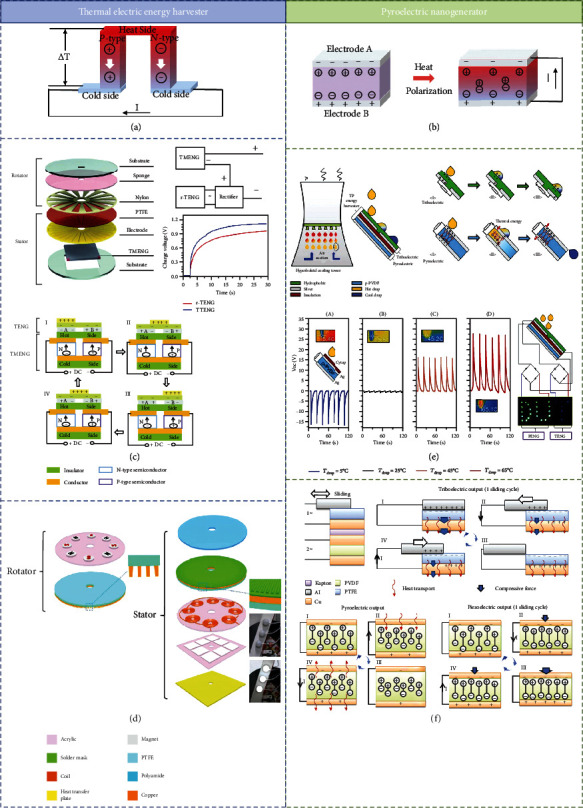
Theoretical comparison and hybrid operation of thermoelectric generator and pyroelectric nanogenerator: (a) the working principle of thermoelectric generator; (b) the working principle of pyroelectric nanogenerator; (c) a 2D rotary triboelectric–thermoelectric hybrid nanogenerator (TTENG) [98] (reproduced with permission. Copyright John Wiley and Sons, 2018); (d) electromagnetic-triboelectric-thermoelectric hybridized nanogenerator [95] (reproduced with permission. Copyright Elsevier, 2016); (e) a triboelectric and pyroelectric hybrid energy harvester for recovering energy from low-grade waste fluids [96] (reproduced with permission. Copyright Elsevier, 2020); (f) triboelectric–pyroelectric–piezoelectric hybrid cell [97] (reproduced with permission. Copyright John Wiley and Sons, 2015).

As a new type of energy conversion material, thermoelectric materials can convert electric energy and heat energy into each other through the microscopic transport process of carrier and phonon [84–86]. The popularization of thermoelectric technology provides a broader idea for improving energy utilization efficiency and alleviating environmental pollution. In 1821, the German physicist Thomas John Seebeck discovered a thermoelectric phenomenon. A closed-loop connecting two conductors of different materials was placed near a compass that could be used to detect micromagnetic fields. When there was a temperature difference between the conductors in the loop, the compass would deflect [87, 88]. As shown in Figure 6(a), for a closed-loop made of two conductor materials of different materials in series, if there is a temperature difference between the two contact points of the material, an electric current would be produced in the closed circuit, which is called as Seebeck effect [89, 90]. From the perspective of energy conversion, the Seebeck effect is a process in which thermal energy is transformed into electric energy. The electromotive force generated by temperature difference is called thermoelectromotive force. The magnitude of thermoelectromotive force is related to the characteristics of the metal material itself and the temperature difference between the two contact points Δ*T* = *T*_1_ − *T*_2_. When the temperature difference is small, the ratio of the thermoelectromotive force to the temperature difference is called the Seebeck coefficient [91], denoted as *S*, in *μ*VK^−1^,

(8) $$\begin{array}{c} V=S_{ab}\left(T_{1}-T_{2}\right). \end{array}$$

For a homogeneous conductor or semiconductor material, *S* represents the capacity of the material to convert thermal energy into electrical energy, i.e.,

(9) $$\begin{array}{c} \underset{∆T\to 0}{\lim}\frac{∆V}{∆T}=S_{ab}. \end{array}$$

For semiconductor materials, when there is a temperature difference between two sides of the material, driven by the external temperature difference, the carrier with higher temperature will diffuse to the low-temperature end. On the other side of the semiconductor, due to the accumulation of carriers, a built-in electric field will form inside the material which will prevent its further diffusion. When the temperature difference is driven and the built-in electric field reaches a balance, a stable thermoelectric electromotive force will be formed in the material.

However, in general, there is basically no large temperature difference at different locations in the same environment. In general, the temperature fluctuates with time. In this case, the Seebeck effect is very small, and the pyroelectric effect of materials is a good way to deal with this situation, as shown in Figure 6(b). In 2012, Yang et al.'s research group published the first article of the pyroelectric nanogenerator [92, 93]. The basic pyroelectric effect plays a major role in many ferroelectric materials. It mainly depends on the free oscillation of the electric dipole inside the material near its own equilibrium axis, and the random oscillation will also increase when the temperature rises. For wurtzite materials such as ZnO and CdS, the secondary pyroelectric effect plays a leading role in the process of converting thermal energy into electrical energy. The main force driving electrons to move is the piezoelectric potential difference induced by thermal deformation in the material cross section.

### 3.2. Hybrid Operation of TENG and Thermoelectric Generator

In application scenarios, TENG must pass through specific material friction to operate. The heat energy produced and wasted during the triboelectric energy generation process limits the output of TENG. As shown in Figure 6(c), Wu et al. designed a triboelectric-thermoelectric hybrid nanogenerator (TTENG) to scavenge the lost thermal energy from the temperature difference induced by rotation-mode TENG friction with mechanical energy from ambient environment [94]. There are two parts of the hybrid generator, a rotator composed of acrylic, sponge, and nylon layer and a stator that consisted of Cu, PTFE, and thermoelectric nanogenerator (TMENG). The TMENG contacts the electrode to harvest the thermal energy from the rotation. The charging voltage of TTENG which is connected in series can reach 1.85 V, which is much higher than that of the only rotation-mode TENG.

There is also a constant loss of thermal energy from friction in the operation of hybrid mechanical energy harvesting device. Especially, EMG has been integrated in the rotation-based TENG to increase the total output while losing more heat energy. To solve the waste of thermal energy, Wang et al. efficiently integrate the TENG and EMG with a thermoelectric generator (ThEG) by cooperative operation, in Figure 6(d) [95]. The EMG part consists of eight magnets (the first layer of rotator) with the magnetic poles in an acrylic disk and eight groups of coils (the third layer of stator). The TENG part consists of the radially arrayed sectors in the second layer of the rotator and stator and the triboelectric material at the first layer of the stator. The ThEG is attached on layer 4 in the stator. This hybridized nanogenerator can produce a constant output voltage of 5 V and a pulsed output current peak of 160 mA, which is applied to the bicycle.

### 3.3. Hybrid Operation of TENG and Pyroelectric Nanogenerator

In general, there is basically no large temperature difference in the ordinary environment. Pyroelectric nanogenerator (PENG) has the potential to convert heat sources with periodized temperature to power. A variety of presentations have been suggested for capturing heat and converting it into electricity. In the last few years, some solid-liquid-based TENGs are demonstrated for collecting the kinetic energy from droplets, flows, water waves, and so on. Besides kinesthetic energy, hot fluids contain thermal energy. Thus, a hybrid triboelectric-pyroelectric nanogenerator (TPENG) was proposed to harvest energy from degrading effluent by Jiang et al.; the device is as shown in Figure 6(e) [96]. The TPENG is composed of a TENG (including the hydrophobic layer and two separated silver electrodes) and the PENG (including the upper silver electrode, the polarized-PVDF, and the below silver electrode). When the temperature of a droplet is lower than PENG device, negative peaks are achieved, while when the temperature of a drop is higher than the PENG, positive peaks are obtained. The PENG can light up “T” (7 LEDs) by connecting a full-wave rectifier with a short duration. The TENG can light up “DU” (21 LEDs) with a longer duration. Because the PVDF layer maintains the pyroelectric and piezoelectric output characteristics at the same time, Zi et al. studied a triboelectric pyroelectric-piezoelectric composite mechanism working device, as shown in Figure 6(f) [97]. The pyroelectric-piezoelectric energy harvester can collect heat generated in friction and pressure produced by the machinery. When this hybrid generator charges the capacitor, this charging effect is twice higher than TENG working alone. This excellent performance makes the device under a complex mechanism provide more possibilities for future self-powered sensors.

## 4. Integration of Mechanical and Solar Energy Harvesters

### 4.1. The Characteristic of Solar Cell

Solar cells are an apparatus that transform solar energy into electrical energy by using the photovoltaic effect. In 1839, French physicist A. E. Becquerel found out the photovoltaic effect. In 1954, Chapin et al. prepared a single-crystalline silicon solar cell for the first time, with a photoelectric conversion efficiency of 6% [99]. Since then, the research of solar cells has begun formally and attracted wide attention. Up to now, it has gone through three generations.

As the first-generation solar cell, crystalline silicon solar cell is the most widely useful because of its high-efficiency performance and excellent stability, although the maximum certified efficiency of monocrystalline and polysilicon solar cells has reached 27.6% and 22.3%, respectively [100, 101]. However, it still faces the problems of complex processes and high material purity. In addition, silicon solar cell devices are brittle and not easy to be made into flexible devices.

Inorganic thin film solar cells as the second generation of solar cells mainly include amorphous silicon (a-Si), gallium arsenide (GaAs), cadmium telluride (CdTe), copper indium gallium selenium (CIGS), and copper zinc tin sulfur (CZTS) solar cells [102–105]. The highest certified efficiency for such solar cells is 30.5% of gallium arsenide. Gallium arsenide solar cells are mainly used in aviation, military industry, and other fields due to the scarcity and high cost of raw materials. Amorphous silicon solar cells account for the highest proportion of thin-film solar cells in the market at present. Although the certified efficiency is only 14%, their large-scale low-temperature preparation and the absence of toxic heavy metals promote their widespread use.

The third generation of new solar cells mainly includes organic, dye sensitization, quantum dot, and perovskite solar cells. Such devices have attracted much attention in modern times caused by their low cost, simple technology, and solvable processing. Among them, the certification efficiency of organic, dye-sensitized, and quantum dot solar cells reached 17.4%, 12.3%, and 16.6%, respectively. Perovskite solar cells are derived from the development of dye-sensitized cells, in ten years, and the certification efficiency of the laboratory has reached 25.2%, surpassing the second generation of inorganic thin film cells and developing very rapidly [14, 106–108].

However, the surface of the solar cell needs a transparent protective layer to preserve solar cell from deterioration, defilement, and impairment. In addition, when the solar cell works, it needs to be exposed to the outside world, and there is the raindrop mechanical energy when it rains, and when it is worn on the human body, there is the loss of mechanical energy in human movement. Because of this, the complementary combination between the solar cell and TENG has attracted more and more attention and has shown great potential for application. Recently, a variety of solar and mechanical energy harvesting hybrid device units have been developed.

### 4.2. Hybrid Operation of Wearable Energy Harvester and Solar Cell

To implement a wearable application of solar cells and meet the requirements by the photoelectric conversion efficiency of the wearable market at the same time, it also needs to develop a light and flexible wearable electronic device which is even stretchable and washable. TENG has developed a number of practical applications in the field of flexible wearable electronic device, and combining it with flexible solar cells can not only significantly increase the total power output but also improve its application in various environmental conditions. This combined energy harvester allows wearable devices to apply in different weather conditions, such as sunny, rainy, and cloudy days, by harvesting mechanical and solar energy.

**Figure 7:**
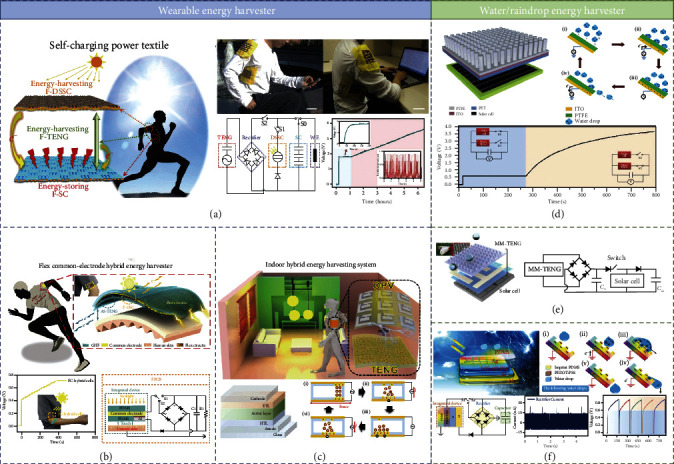
Hybrid operation of water energy harvester and water/raindrop energy harvester: (a) self-charging power textile [109]; (b) flex common-electrode hybrid energy harvester [110] (reproduced with permission. Copyright Elsevier, 2020); (c) indoor hybrid energy harvesting system [111] (reproduced with permission. Copyright Elsevier, 2020); (d) micropyramidal silicon-based hybrid cell [112] (reproduced with permission. Copyright Elsevier, 2014); (e) self-cleaning moth's eye mimicking TENG-based hybrid energy harvester [113] (reproduced with permission. Copyright Elsevier, 2019); (f) shared mutual function electrode-based integrated system [114] (reproduced with permission. Copyright American Chemical Society, 2018).

Wen et al. designed a self-powered hybrid fiber-based nanogenerator, solar cells, and supercapacitors [109] (Figure 7(a)). There are three kinds of functional devices. For the fiber-shaped dye-sensitized solar cell (F-DSSCs), N719 dye is used as sensitizer, a Ti wire with TiO_2_ nanotube is used as a working electrode, a carbon fiber/Pt is used as a a counter electrode, and I^−^/I^3−^ is used as a the based electrolyte. Cu-plated EVA tube serve not only as scaffold for solar cell fabrication but also as one electrode for F-TENG. For F-SCs, the EVA tubes were covered with Cu, which works as the scaffold for F-SC fabrication and one electrode for F-TENG. The F-TENG is the contact-separation mode. A tester who wore textile, harvests solar energy and mechanical energy working in the tester's daily activities. By the equivalent circuit, the F-SCs achieve 1.8 V because of the low output voltage of the F-DSSCs in the blue area. It can be powered to a higher voltage by the F-TENGs in a red-shaded area.

Current approaches to miniaturing flexible miniaturized energy harvesting systems can be achieved by coating polymer substrates directly with multiple functional layers. As shown in Figure 7(b), Ren et al. used a groove-shaped micro-/nanostructured haze thin film (GHF) on the surface of F-OSC and AS-TENG to achieve excellent optical properties, large surface area, and superhydrophobic performance [110]. A common electrode (PEN/ITO) that acts as a highlight of the structure design makes the whole device integrated optimization. The voltage of capacitor could achieve 0.7 V, which breaks the limit of F-OSC. In addition, due to the adjustable absorption spectrum of the active materials in OSCs, which can be easily matched with the indoor light sources. Considering its capability and feasibility to obtain indoor energy from human activities, the TENG seems to be suitable for indoor applications in combination with OSCs. In Figure 7(c), Jung et al. consider the need for indoor energy harvesting and integrate the 3D Cu ball-based TENG and OSCs as a hybrid system [111].

### 4.3. Hybrid Operation of Water/Raindrop Energy Harvester and Solar Cell

Rainy days greatly reduce the sun's radiation, and many solar cells need a protective film to keep water vapor out. Thus, improving energy harvesting efficiency on rainy days has led to the development of a variety of solar and rain energy hybrid device units. TENG has proved to be a viable solution for collecting mechanical energy of water/raindrop due to the presence of contact electrification at the solid-liquid interface. Therefore, TENG and solar cell integrated devices are particularly suitable for working in complex outdoor environments.

In Figure 7(d), attributed to the contact electrification and electrostatic induction in the process of raindrop, Zheng et al. processed a transparent TENG (PTFE/ITO/PET) to protect the silicon-based solar cell from environmental damages, which can harvest energy from raindrop. In practical applications, TENG and Si solar cells are used in parallel to charge commercial capacitors [112]. The Si solar cell can only charge the 33 *μ*F capacitor to 0.6 V, but the rectified TENG under a water dripping rate of 0.116 mL/s is used to charge the same capacitor to 3.5 V for 530 s. This confirms that the hybrid energy cell can provide electric output in different weather conditions. Considering that the transparent property of TENG device affects the optical characteristics of solar cell, Yoo et al. reported a moth's eye mimicking TENG (MM-TENG) with high transmittance (91% for visible light), which is hybrid with a conventional solar cell [113]. At the same time, through a novel electric circuit for effective management in a hybrid energy harvester, environmental energy is properly collected and energy efficiency is improved in Figure 7(e).

In line with hybrid energy harvesters, it is not only a simple superposition of different energy devices but also a composite structure to reduce the cost and volume of devices. Liu et al. demonstrated a heterojunction silicon solar cell integrated with TENG by a mutual electrode of a poly(3,4- ethylene dioxythiophene):poly(styrene sulfonate) (PEDOT:PSS) film [114]. For the structural design of a mutual electrode, the special circuit of the integrated device is as shown in Figure 7(f). The current output of the TENG after rectification is 24.2 nA. When the solar cell is charging the capacitor, the highest voltage is 0.6 V, and the voltage increases to 0.9 V by the high voltage of TENG. This integrated system successfully converts both solar energy and raindrop energy into electrical power.

## 5. Converting Hybrid Energy to Hydrogen Energy

### 5.1. The Characteristic of Electrochemical Water Splitting and Photoelectrochemical (PEC) Water Splitting Combined with TENG

As an energy transformer, TENG can be combined with electrochemical processes to produce H_2_ by water splitting without the use of external power source, which contains direct drive electrolytic water and photoelectrochemical (PEC) water splitting, respectively [115]. Environmental mechanical energy could be fully transformed into chemical energy, such as solar energy, wind energy, and flowing kinetic energy. Up to now, several self-powered electrochemical systems have also been manufactured considering the universal applicability of mechanical energy for specific practical applications. Thus, how to utilize the TENG to trigger a self-powered water splitting process has always been the focus of our attention. The objective of this part focuses on the fundamental principle, updated progress, and potential applications of TENG-based self-powered water splitting.

**Figure 8:**
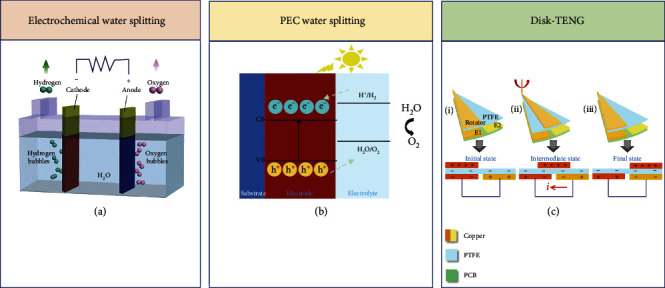
Schematic illustration of (a) the electrochemical water process [116] and (b) PEC water splitting [117] (reproduced with permission. Copyright Royal Society of Chemistry, 2015) (reproduced with permission. Copyright Royal Society of John Wiley and Sons, 2020); (c) schematics of operating mechanism of the disk-TENG [118] (reproduced with permission. Copyright Royal Society of American Chemical Society, 2018).

Figure 8(a) schematically illustrates the process of electrolytic water [116]. When the direct current is applied to the electrode, the H^+^ in the electrolyte migrate to the cathode driven by the external electric field and get electrons to generate H_2_, Simultaneously, the OH^−^ migrate to the anode for water oxidation to produce O_2_. It is worth mentioning that the minimum voltage for electrolytic water is ~1.23 V. For the PEC water splitting process, the conduction band (CB) potential of photoelectrode in energy is lower than hydrogen evolution potential (Figure 8(b)) [117]. The potential of the valance band (VB) is higher than that of oxygen evolution potential. Under illumination, the electrons on the VB are excited to jump to CB and generated electron-hole pairs. When the applied voltage is up to the onset potential for PEC water splitting, the electrons migrate to counter electrode driven by the external electric field and to generate hydrogen, while the holes own high oxidizing ability and will migrate to the interface to oxidize water for producing oxygen. Compared with other TENG structures, the rotary-disk TENG has the advantage of large power output and can provide sufficient external power for the water splitting process. Therefore, this structure is mostly used in the self-driven water splitting system. Its working principle is shown in Figure 8(c) [118]. The moved charge flow between two triboelectric materials with the opposite polarity of TENG leads to triboelectrification and electrostatic induction, and alternating flow of electrons between electrodes will push electrons around.

### 5.2. Hybrid Operation of Electrochemical Water Splitting and TENG

**Figure 9:**
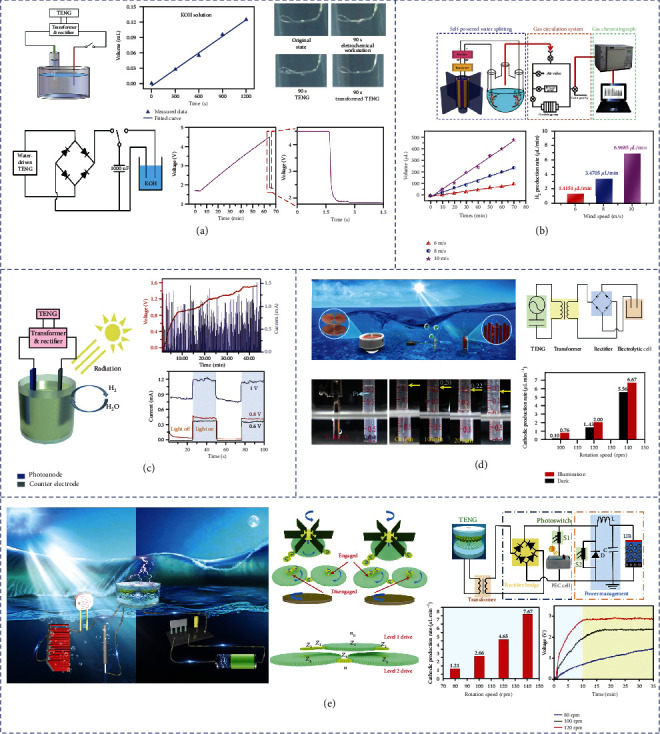
Self-powered water splitting: (a) self-powered water splitting using flowing kinetic energy [119] (reproduced with permission. Copyright Royal Society of John Wiley and Sons, 2014); (b) wind energy harvester based on coaxial rotatory freestanding triboelectric nanogenerators for self-powered water splitting [120] (reproduced with permission. Copyright Royal Society of Elsevier, 2018) (c) boosting photoelectrochemical water splitting by TENG-charged Li-ion battery [121] (reproduced with permission. Copyright Royal Society of John Wiley and Sons, 2017); (d) TENG-driven self-powered photoelectrochemical water splitting based on hematite photoanodes [118] (reproduced with permission. Copyright Royal Society of American Chemical Society, 2018) (e) Blue energy collection toward all-hour self-powered chemical energy conversion [117] (reproduced with permission. Copyright Royal Society of John Wiley and Sons, 2020).

Tang et al. developed a fully self-driven water splitting organization that could realize water splitting by coupling a TENG and a water splitting part based on the flowing kinetic energy, as shown in Figure 9(a) [119]. In the schematic diagram, we can see that when the TENG is connected to the water splitting unit, the output of the TENG is firstly transformed and rectified, the Pt is selected as the working electrode of the electrolysis, and the 30% (w.t) KOH solution is used as the electrolyte. For collecting the H_2_, a tube is inserted into the cathode. The volume of the generated H_2_ and splitting time show a linear relationship with a slope of 6.25 × 10^−3^ mL min^−1^ at 600 rpm. In addition, when the pure water was selected as electrolyte instead of KOH solution, the TENG-driven system produces four H_2_ bubbles in 90 s with a voltage of 10 V that is four times the electrochemical workstation. This indicates that the high voltage of the TENG is capable of prevailing over the high load, which is also the advantage of TENG in pure water electrolysis. With the aim of showing the splitting process in distinct, a 1000 *μ*F capacitor is working as a power store. Among the whole process, electricity is firstly stored in the capacitor and then used for splitting. The output of the capacitor rises from 1.7 V to 4.5 V within 60 minutes. After connecting to the water splitting system, the voltage quickly discharged to 1.8 V, which is sufficient as the decomposition voltage of KOH solution. Besides, wind energy is another renewable energy source with the most promising development, utilized prospects, and mature technology. It is an economic, sustainable, and environmentally friendly route for converting wind energy into H_2_. Ren *et al*. have reported a coaxial rotatory freestanding TENG (CRF-TENG) wind energy harvester and present a completely self-powered water splitting system for H_2_ production, as shown in Figure 9(b) [120]. This H_2_ generation system consists of three parts: self-powered water splitting unit, gas circulation system, and gas chromatograph. Being connected to a transformer and a rectifier bridge, the CRF-TENG wind energy harvester is then connected with an electrolytic bath to form a self-powered water splitting unit. The produced hydrogen is injected into the gas chromatograph through the gas circulation system and then quantitatively analyzed. This method of evaluating the hydrogen evolution rate is more accurate than other methods using the gas collection tube. As time goes on, the volume of the produced hydrogen gradually increases. The linear relationship between gas production and time shows that the slope rises increasing with wind speed. When the wind speed is 6 m/s, 8 m/s, and 10 m/s, the hydrogen evolution rate can be calculated to 1.4151 *μ*L min^−1^, 3.4705 *μ*L min^−1^, and 6.9685 *μ*L min^−1^, respectively.

### 5.3. Hybrid Operation of PEC Water Splitting and TENG

In addition to directly driving electrolytic water, PEC processes combined with TENG to produce H_2_ by water splitting are an ideal approach, since the direct electrolysis of water requires a higher external bias than the photoelectrolysis process. The voltage output of TENG with peak output characteristics does not always keep at the peak value, and the majority of the voltage output is typically below the peak value for direct electrolysis. With the main low working voltage, photoelectrochemical (PEC) water splitting can play a major role and the sunlight effect is very important to accelerate the reaction. Thus, the PEC water splitting has great advantages over the direct hydrolysis when using TENG. Actually, direct electrolysis and PEC water splitting simultaneously occur at a high rotation speed. This becomes an effective strategy for obtaining hydrogen energy through PEC water splitting to convert solar and mechanical energy. Li et al. developed a new type TENG-PEC-based hybrid cell using TiO_2_ as photoanode, which enables researchers to realize water splitting through coupling electrolysis and PEC effect. The water splitting of PEC is significantly boosted by a TENG-charged Li-ion battery, as shown in Figure 9(c) [121]. The basic principle of the TENG-PEC hybrid cells is that a flexible TENG collects and transforms mechanical energy into electric current, which boosts the PEC water splitting via the charged Li-ion battery. A typical design of flexible TENG works under the contact-separation process between PTFE and ITO. After transformation and rectification, the electricity generated by TENG can be stored in a Li-ion battery. The TENG can charge the Li-ion battery to 1.5 V within 42 minutes driven by a linear motor with a force of 60 N. The photocurrent of Li-ion battery charged by TENG is raised to 1.66 mA cm^−2^. It is increased to 0.5 mA when the working electrode area is 0.3 cm^2^ with a bias of 0.8 V while the current in the dark remains zero. However, when the bias is 1 V, a clear electrolytic process will be still presented in the absence of light and the photocurrent will reach 0.82 mA at the moment. The obvious photoresponse with light is about 0.4 mA. The above results show that both electrolysis and PEC water splitting coexist when the Li-ion battery is embedded in the circuit, which is totally diverse from the conventional PEC cells. Although the combination of TENG, Li-ion battery, and PEC water splitting is a wonderful design, where the PEC water splitting was distinctly boosted by a TENG-charged Li-ion battery, such a two-step process still has some significant drawbacks. In other words, the energy generated by TENG is firstly stored in a Li-ion battery; then, the TENG's electrical energy is stored in a battery to drive PEC. There are two steps of energy conversion and release in this process, which involves inevitable energy loss during the charging and discharging process. In the energy harvesting and conversion system, the inevitable energy loss greatly affects the energy conversion efficiency. Since the Li-ion battery current output is the high instantaneous current which is unstable, the output power of the Li-ion battery will decrease as the discharge time, leading to the deceased H_2_ generation rate with time. It is impossible to obtain stable and continuous hydrogen production. And in the PEC technique, another critical component is the photoelectrode, which should exhibit excellent photoelectric performance. To make better utilization of marine mechanical energy and improve energy conversion efficiency, Wei et al. successfully fabricated a self-powered PEC water splitting system based on photoanodes driven by an RD-TENG to produce H_2_, as illustrated in Figure 9(d) [118]. Since TENG can generate electricity easily and produce necessary external bias for self-hydrolysis, an RD-TENG was applied to the self-powered PEC water splitting system. To measure the output performance, a programmable rotary motor is used and connected to the turntable simulating water flowing to control the rotation speed. The open-circuit voltage and short-circuit current at different speeds of rotation after transformation have been measured. With the increase of rotation speed, both of *V*_OC_ and *I*_SC_ are increased at the same time. After transformation and rectification, it is more than enough to provide the voltage required for photoelectrolysis of water. And after testing and characterizations, it is confirmed that Ti-Fe_2_O_3_ is an excellent and stable photoelectrolytic material. In order to evaluate the capacity of H_2_ production of the self-powered PEC water splitting system, the H_2_ generation rate is measured at three different speeds under darkness or light. At relatively low rotation speeds, the rates of H_2_ production under illumination or darkness are very low. When the rotation speed reaches 140 rpm, the rate of H_2_ production will be increased to 5.56 and 6.67 *μ*L min^−1^ before and after illuminating, respectively. Herein, such a self-powered PEC water splitting system enables to convert both solar energy and marine mechanical energy in the form of hydrogen energy.

The direction of water flow in nature is mostly disorderly, TENG cannot fully utilize the deep ocean mechanical energy of the environment if it is used only for PEC process under light illumination. Due to the peak output characteristics of TENGs, the output cannot always keep at the best value to make sure the average output from TENGs is typically in the range for PEC water splitting. Using the generator on the PEC water alone will make most of the energy unable to be converted, which is limited by weather conditions. Actually, the efficiency of PEC is relatively low. Another real problem is that the energy transmission from the deep ocean is very difficult and a suitable energy conversion method for the use of TENGs in the deep ocean is urgent. The above problems all make it necessary to propose an efficient and all-hour energy harvesting and conversion system to supplement the actual situation. Zhai et al. designed a GUA-TENG to collect water flow kinetic energy to drive a Ti-PEC under illumination to produce H_2_ [117]. Moreover, under the situation without enough sunlight, the self-powered system can be automatically switched to another working state to charge a C_O_-LIB for powering a small device (Figure 9(e)). The whole system can convert the blue energy to either hydrogen energy or electrical energy for easy storage and transmission, which not only achieves effective utilization of clean ocean energy but also improves energy conversion. The gear-driven unidirectional acceleration TENG (GUA-TENG) system can harvest water flow kinetic energy efficient by high-ratio synergy pairs of gears and pinions, which can transform the disordered low-frequency ocean flowing into one-way flow energy and increase the rotation speed by 25 times simultaneously. When testing electrical output in the laboratory, they replace the ocean water flow kinetic energy with a motor to drive the disk directly. From the test results, the excellent photoelectric performance of Ti-Fe_2_O_3_/FeNiOOH can be obtained. Meanwhile, the Li-ion battery also has excellent stability. The excellent performance of both the Ti-Fe_2_O_3_/FeNiOOH and Li-ion batteries indicate the potential in blue energy conversion systems. In the circuit diagram of the whole system, under illumination, the resistance of S1 is 0, the system performs the Ti-PEC process. Under dark conditions, the resistance of S2 is 0, and the system charges the C_O_-LIB. To improve energy storage conversion efficiency, a power management module is used for C_O_-LIB charging. The state of S1 and S2 is controlled autonomously by the light condition. Therefore, the mechanical energy and solar energy are both collected and converted into chemical energy in the form of hydrogen energy. The mechanical energy collected by the GUA-TENG collected by C_O_-LIB can be converted into electricity. The whole system achieves high-efficiency all-time self-powered chemical energy conversion. Under illumination, when the rotation speed is at 120 rpm, the H_2_ generation rate can reach 4.65 *μ*L/min, which also proves the effective working state of the photoelectrodes. Under dark conditions, when the speed is 100 rpm, the voltage can reach 2.35 V within 15 min. With the increase of speed, the charging time is shortened. It takes only 10 minutes to charge to 2.75 V at 120 rpm. It also shows that the whole system has good electrochemical performance in storing electrical energy generated from blue energy, and the energy conversion efficiency of the whole system is to be 2.29%. The whole system achieves high-efficiency all-time self-powered chemical energy conversion.

## 6. Outlook and Summary

With the increasing energy crisis and the expanding popularity of personal portable electronic devices, the research and development of new energy have been widely concerned by researchers all over the world. The main characteristics of modern new energy are green, safe, reliable, and low cost. As an effective mechanical energy harvesting device, the new development of TENG can convert the waste mechanical energy into valuable electrical energy by the coupling of triboelectrification and electrostatic induction [5–7], because the TENG has advantages of high-efficiency, lightweight, low cost, and environmental protection, in the collection of low-frequency random mechanical energy but has disadvantages of low current output and not easily stored in large capacitors. Combining with other generators that harvest various energies around the environment and constructing hybridized TENG are a solution to improve energy efficiency and power the vast Internet of Things, infinite sensor network, and wearable electronic devices.

This review starts from the mechanism of several energy harvesting devices to analysis of their applicable scenarios, from energy complementation to the design of composite structures. And the multifunctional application of hybrid energy devices can adapt to different needs. By integrating TENG with EMG, the range of mechanical energy collection (from low frequency to high frequency and from small amplitude to large amplitude) can be widened to improve the energy efficiency of mechanical energy harvesting, and due to the large devices of EMGs, the hybrid devices are suitable for harvesting wind and water wave energy. The integration of TENG and PENG reduces the loss of mechanical energy in the vertical direction, and the output is improved as a wearable power supply device because the structure and materials of both kinds of devices can be designed as flexible. The combination of TENG with thermal energy harvesters obtains heat energy from the friction of inherent sliding motions of TENG and wasted heat energy in production and life. The hybrid energy cell of TENG and solar cell compensates the working time so that it can supply power to the device when there is no sunlight, and improves the maximum voltage when charging capacity. Due to the high power output of the TENG, it can provide sufficient external power for the water splitting process. For the TENG-driven water splitting system, environmental mechanical energy can be fully converted into chemical energy.

**Figure 10:**
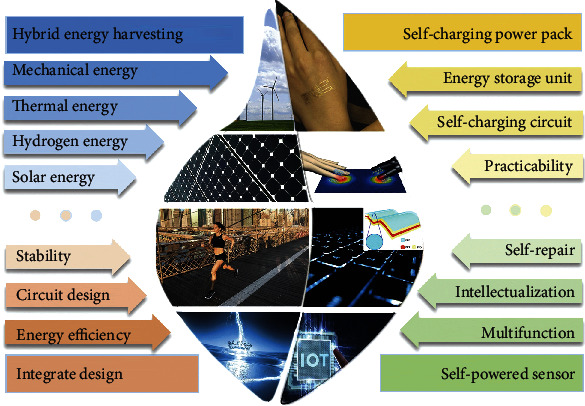
Prospective of hybrid triboelectric nanogenerators, include hybrid energy harvesting, integrated design, self-charging power pack, and self-powered sensor [122–124] (reproduced with permission. Copyright Royal Society of John Wiley and Sons, 2016; reproduced with permission. Copyright Royal Society of Elsevier, 2016; reproduced with permission. Copyright Royal Society of John Wiley and Sons, 2015).

The device with a single energy exchange mechanism is limited by its conversion efficiency and cannot achieve the maximum conversion of energy. As shown in Figure 10, the hybrid generator is highly integrated with different energy harvesting modes to improve the output performance and has a wide application prospect, including the self-charging power pack and self-powered sensor. As for implementing these practical applications, there are still some problems with hybrid energy harvesters based on TENG that need to be solved:

1. Hybrid energy harvesting: the integration of various kinds of energy is mainly a compound of two kinds of energy; few have a combination of three or more energy sources. Subject to the limitations of functional materials themselves, various collection devices need to improve the characteristics of the materials themselves to enhance the output indicators
2. Integrate design: at present, the composition of hybrid energy mechanisms is mainly reflected in a simple stack of different energy harvesters. Thus, the energy efficiency of the hybrid system can be improved through the reasonable layout of the placement positions of different energy units. However, there is little research on the complex energy management circuit. Because different energy harvesting units have different system resistance and response characteristics, the single-strategy energy management mode or management chip is not applicable. At the same time, the stability of every energy unit in the hybrid system and the whole structure needs to be optimized to meet harsh environments and long working hours
3. Self-charging power pack: although the hybrid energy harvesting can improve the energy efficiency to a certain extent, the energy scale of TENG still belongs to the category of microenergy, and minimizing the power consumption of the back-end management circuit is the long-term pursuit goal in this field. Energy storage devices matching different hybrid energy devices are also less studied, such as batteries and supercapacitors. Wearable electronic devices have increasing application potential in the field of the Internet of Things, but the limitation of traditional power sources has been a huge challenge. To meet this challenge, integrating wearable energy harvesting device and energy storage device into the self-powered device is a promising solution. Thus, as for a wearable self-charging power pack, the hybrid energy system needs to be designed as a flexible, light, washable, and wearable power pack
4. Self-powered sensor: because the different energy collection devices themselves are sensitive to different signals, hybrid self-powered sensors are appropriate for multisignal recognition. Multifunctional self-powered sensors based on hybrid energy devices avoid signal crossing and can detect multiple signals at the same time, which is a reliable way for future integrated sensor networks. However, in order to meet the practical application challenges, the sensors are facing the problem of damage and unable to work for a long time. There has been no progress in the self-repair function of the multifunction sensor, and this problem needs to be overcome. Intelligence is the future development trend of sensors. The sensor integrated microprocessor can learn, judge, and process signals; its ability determines that the intelligent sensor also has higher precision and resolution, higher stability and reliability, and better adaptability
